# Investigating the characteristics of mild intervertebral disc degeneration at various age stages using single-cell genomics

**DOI:** 10.3389/fcell.2024.1409287

**Published:** 2024-07-02

**Authors:** Pengcheng Liu, Xiang Ren, Beiting Zhang, Song Guo, Qiang Fu

**Affiliations:** ^1^ Department of Orthopedics Surgery, Shanghai General Hospital, Shanghai Jiao Tong University, Shanghai, China; ^2^ Interdisciplinary Research Center on Biology and Chemistry, Shanghai Institute of Organic Chemistry, Chinese Academy of Sciences, Shanghai, China

**Keywords:** intervertebral disc degeneration, single-cell genomics, nucleus pulposus, hypoxia-inducible factor 1 alpha, dendritic cells

## Abstract

**Introduction:** Intervertebral disc degeneration often occurs in the elderly population, but in recent years, there has been an increasing incidence of disc degeneration in younger individuals, primarily with mild degeneration.

**Methods:** In order to explore the underlying mechanisms of disc degeneration in both young and aging individuals, we collected four types of nucleus pulposus (NP) single-cell sequencing samples for analysis based on Pfirrmann grading: normal-young (NY) (Grade I), normal-old (NO) (Grade I), mild degenerative-young (MY) (Grade II-III), and mild degenerative-old (MO) (Grade II-III).

**Results:** We found that most NP cells in NO and MY samples exhibited oxidative stress, which may be important pathogenic factors in NO and MY groups. On the other hand, NP cells in MO group exhibited endoplasmic reticulum stress. In terms of inflammation, myeloid cells were mainly present in the degenerative group, with the MY group showing a stronger immune response compared to the MO group. Interestingly, dendritic cells in the myeloid lineage played a critical role in the process of mild degeneration.

**Discussion:** Our study investigated the molecular mechanisms of intervertebral disc degeneration from an age perspective, providing insights for improving treatment strategies for patients with disc degeneration at different age groups.

## Introduction

Low back pain (LBP) is a common clinical condition that has become increasingly prevalent. Statistical reports indicate that the global incidence rate of LBP ranges between 13.1% and 28.5% (Maher et al., 2017). Among the various identified factors, intervertebral disc degeneration (IVD) stands out as a primary contributor to LBP, emerging as a leading cause of disability worldwide and exerting significant strain on healthcare systems and economies (Chou, 2021; Knezevic et al., 2021; Yang et al., 2024). To date, most treatments of IVD degeneration are limited to invasive surgical interventions, such as disc replacement and spinal fusion, or pain management, which often fail to restore the integrity and normal physiological function of degenerated intervertebral discs (Knezevic et al., 2017; Meisel et al., 2019).

The intervertebral disc, a sealed structure situated between the vertebral bodies in the human spine, comprises cartilage plates, fibrous rings, and a nucleus pulposus (NP) (Gantenbein et al., 2023), which help sustain the natural flexibility and normal biomechanical functioning of the spine. Researchers have established that dysfunction in any component of the IVD leads to intervertebral disc degeneration (IVDD), with NP tissue degeneration recognized as the primary cause (Lama et al., 2022). IVDD is a multifactorial process with aging being a significant risk factor (Ngo et al., 2017; Iwakiri et al., 2023; Novais et al., 2024). However, disc degeneration can also manifest independently of the aging process, making it challenging to distinguish between physiological disc aging and degeneration (Soufi et al., 2023).

In recent years, single-cell RNA sequencing (scRNA-seq) has gained prominence as a powerful method for evaluating single-cell gene expression, identifying heterogeneous cell subpopulations within tissues, unveiling rare subpopulations, delineating cellular heterogeneity, discovering new markers, and predicting developmental trajectories. It offers insights into physiological and pathological processes and has been increasingly applied in IVD research. Several studies have identified novel phenotypic biomarkers of NP cells, significantly enhancing our understanding of NP cell biology and development (Fernandes et al., 2020; Ling et al., 2021; Panebianco et al., 2021; Zhang et al., 2021; Cherif et al., 2022; Han et al., 2022; RNA, 2022; Guo et al., 2023a; Chen et al., 2024). However, the heterogeneity of IVDD among young and elderly individuals remains to be fully elucidated.

To address this gap, we collected four types of nucleus pulposus (NP) single-cell sequencing data for analysis based on Pfirrmann grading: normal-young (NY) (Grade I), normal-old (NO) (Grade I), mild degenerative-young (MY) (Grade II-III), and mild degenerative-old (MO) (Grade II-III). We conducted in-depth gene ontology term enrichment and pathway analyses and predicted developmental trajectories for each type. Furthermore, we validated our findings through histology and immunofluorescence. The objective of this study was to explore the underlying mechanisms of disc degeneration in both young and aging individuals. These investigations aim to clarify the mechanisms of IVDD from an age perspective and offer insights for improving treatment strategies for patients with disc degeneration across different age groups.

## Methods

### Patients and tissue samples

The Institutional Review Board of the Shanghai General Hospital approved this study. Degenerative NP tissues were collected from patients undergoing surgery due to IVDD (Supplementary Figure S6). Healthy tissues were collected from patients who underwent surgery for idiopathic scoliosis or vertebral fracture. All samples were trimmed in order to preserve the nucleus pulposus tissue while removing the fibrous annulus. Before sample collection, the degree of IVD degeneration was evaluated according to the Pfirrmann grading system (Pfirrmann et al., 2001) (Supplementary Table S1).

### Histology and immunofluorescence

IVD samples from the sample were fixed for 48 h using 4% paraformaldehyde (PFA), dehydrated and embedded in paraffin. Five-micron thick sections were cut from the paraffin blocks. For histological assessment, the paraffin sections were de-paraffinized in graded xylene, rehydrated in graded alcohol solutions, and washed and stained with hematoxylin and eosin (H&E) staining (Servicebio, Wuhan, China) per the manufacturer’s protocols.

For immunofluorescence staining, prepared paraffin sections were incubated in antigen retrieval buffer (Roche Diagnostics) at 37°C for 30 min. After cooling to RT, the sections were washed with PBS three times for 5 min each, followed by permeabilized for 10 min with 0.1% Triton X-100. Then the sections were washed and blocked for 1 h with 1% PBS/BSA and incubated with primary antibodies (anti-SOD2, anti-TOMM20, anti-CD68, anti-Collegen2, anti-HIF1α) in PBS/BSA at 4°C overnight. Afterwards secondary antibodies conjugated with Alexa Fluor 488 and/or 594 were used to incubate cells for 1 h at room temperature. The nucleus was labelled with 10 μg/mL DAPI solution. Pictures were captured using section scanner (Pannoramic MIDI). Images were analyzed using CaseViewer software (C.V2.4).

### Single-cell RNA sequencing

Single cell sequencing data was downloaded from NCBI GEO with accession number GSE233666 (Guo et al., 2023b), GSE205535 (RNA, 2022), GSE189916 (Jiang et al., 2022) and CNGBdb with accession number CNP0002664 (Han et al., 2022). In short, the downloaded data was used to construct a Seurat object using the CreateSeuratObject function in Seurat package (v4.3.0) (Stuart et al., 2019), and the DoubletFinder package (v2.0.3) (McGinnis et al., 2019) was utilized to remove doublets. Cells were filtered by gene counts between 200 and 7,500 and UMI counts over 1,000. Number of genes detected per UMI are higher than 0.8 to remove the outlier cells which have less complex RNA species like red blood cells. Cells with over 15% mitochondrial content were removed. For dimension reduction and clustering, we utilized functions from Seurat package. To ensure uniform gene expression measurement, normalization and scaling were performed using NormalizeData and ScaleData methods. We conducted PCA on the top 2,000 variable genes, which were identified using FindVariableFeatures. The batch effects were eliminated in all datasets using the default parameters of the Harmony package (v0.1.1) (Korsunsky et al., 2019). The cells were subsequently classified into clusters using FindNeighbors and FindClusters with the top 20 principle components and a resolution parameter of 0.1. The DimPlot function was employed to generate a 2D Uniform Manifold Approximation and Projection (UMAP) plot using the “UMAP” reduction method. Cell-type identity of each cluster was determined through manual annotation using the expression of canonical markers. DotPlot, FeaturePlot, and Vlnplot functions were utilized to generate dot plots, feature plots, and violin plots, respectively, to visualize the expression of markers used for cell type identification. Differentially expressed genes (DEGs) within each celltype were identified using Seurat’s FindMarkers function, employing a Wilcoxon likelihood ratio test with default parameters. Genes expressed in over 10% of cells within a cluster and exhibiting an average log-fold change greater than 0.25 were considered as DEGs. The GO enrichment analysis with DEGs was conducted using the clusterProfiler package. The strength of signaling pathways was calculated using the AUCell package (v1.20.2) (Deng et al., 2021).

### Pseudotime analysis

To determine the cell lineage of subclusters, Slingshot (Street et al., 2018) was used to find lineage by fitting minimum spanning trees based on clusters through the getLineages function. After constructing the smooth lineages, we inferred the pseudo time of each cell by fitting the principal curves through the getCurves function.

### Cell communication analysis

The cell-cell communication network was identified by the CellChat R package as previously described (Jin et al., 2021). The cellular communications between different groups in IVD were analyzed. Briefly, we construct cellchat object base on the expression matrix and meta information from Seurat by using createCellChat function. Then, merge cellchat object of different grade sample by mergeCellChat function. After these, we use compareInteractions to compare different interactions between different grades and net Visual_diffInteraction function to visualize results.

CellChat (v1.1.3) was applied for ligand–receptor analysis. The normalized counts and cell-type annotations for each cell were imputed into CellChat to determine the potential ligand–receptor pairs. Interaction pairs with *p*-value > 0.05 were filtered out from further analysis. The interaction of myeloid cells and NP cells was analyzed. Selected specific pairs were plotted in CellChat with default parameters.

## Results

### Single-cell sequencing analysis reveals heterogeneity of intervertebral disc cells during degeneration and aging processes

**FIGURE 1:**
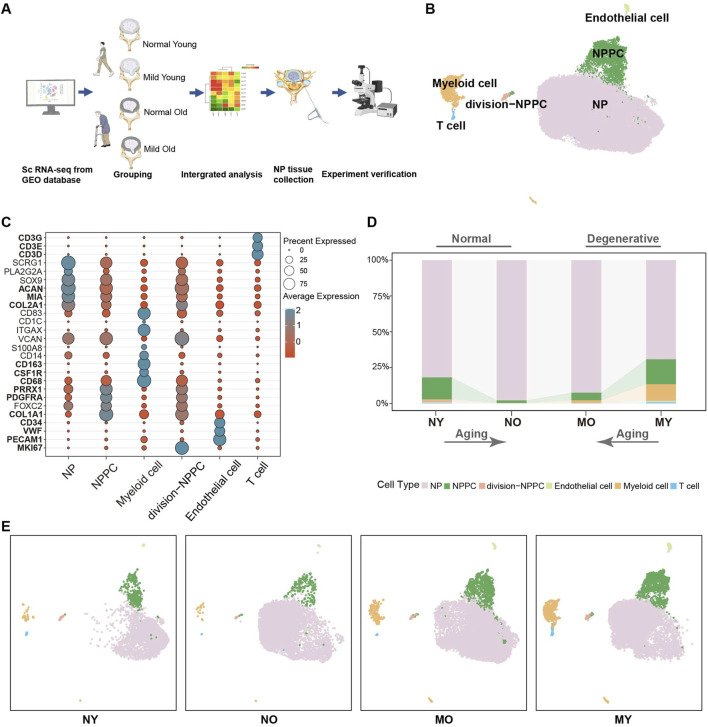
Single-cell RNA sequencing analysis of human IVD cells. **(A)** Schematic workflow of sc-RNA sequencing analysis and experimental validation. **(B)** Uni-form Mani-fold Approximation and Projection (UMAP) showing the single-cell transcriptomic landscape of human IVD cells. Six clusters were visualized. **(C)** Dot plot of marker genes for distinct cell types. Color scale indicates the mean of normalized expression of marker genes in each cell type, and dot size is proportional to the percentage of cells within each cell cluster expressing the marker genes. **(D)** Distribution of cell clusters (defined in B) in different groups. NY, normal-young (Grade I), NO, normal-old (Grade I), MO, mild degenerative-young (Grade II-III), MY, mild degenerative-old (Grade II-III). **(E)** Same UMAP visualization as the one in B but split by different groups.

To investigate the transcriptomic changes in the intervertebral disc during normal and mild degeneration in both young and elderly individuals, capturing potential initiating factors of intervertebral disc degeneration (IDD), we collected a total of 10 nucleus pulposus (NP) single-cell sequencing datasets (Supplementary Table S2) based on the Pfirrmann grading. The datasets included samples from normal-young (Grade I, NY), normal-old (Grade I, NO), mild degenerative-young (Grade II-III, MY), and mild degenerative-old (Grade II-III, MO). After quality control processes such as filtering out low-quality cells, doublets, and batch effects, a total of 50,077 high-quality NP cells were obtained for downstream analysis (Figure 1A).

Based on the classical markers, we have initially divided the overall cells into six celltypes (Figures 1B, C), which are NP cell (*COL2A1, MIA, ACAN*), nucleus pulposus progenitor cell (NPPC) (*COL1A1, PDGFRA, PRRX1*), division-NPPC (*MKI67*), myeloid cell (*CD68, CSF1R, CD163*), endothelial cell (*PECAM1, VWF, CD34*), and T cell (*CD3D, CD3E, CD3G*). We found that NP cell were the main components in all groups, with no significant changes between groups. The proportion of NPPC decreased with aging in both normal and degenerated samples, while immune cells were mainly present in degenerated samples. Moreover, MY group had higher levels of immune cell infiltration than the MO group, suggesting that samples from younger patients exhibited more immune cell infiltration in mild degenerative disc disease (Figures 1D, E).

### Oxidative stress is an important pathogenic factor in the NP cell of both NO and MY groups

**FIGURE 2:**
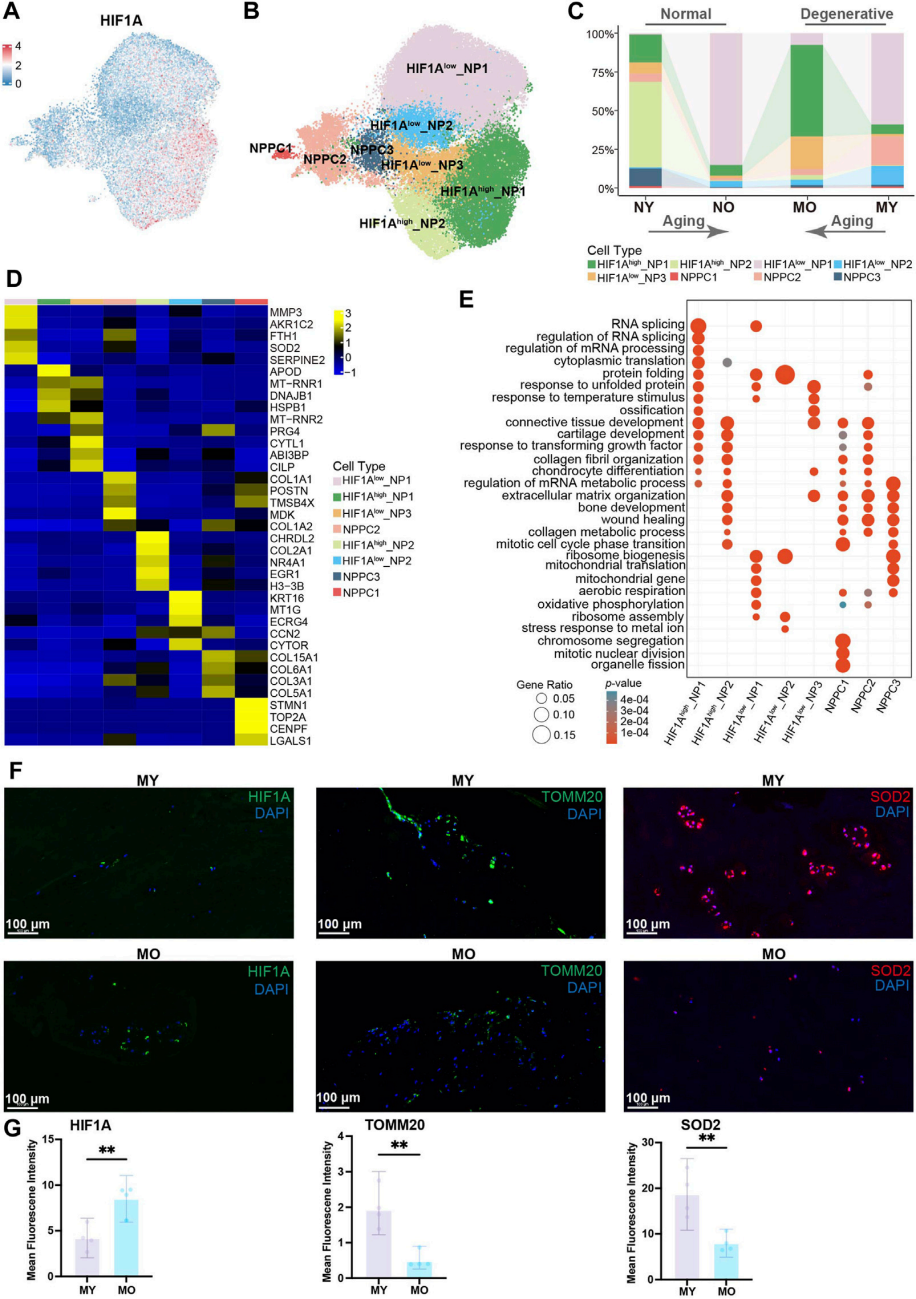
Identification of NP cell subpopulations and their distribution in different groups. **(A)** Expression of *HIF1A* in human NP cells (including NP cell, NPPC, division-NPPC) projected onto UMAP visualization. NPPC, nucleus pulposus progenitor cell. **(B)** UMAP plot showing the single-cell transcriptomic landscape of human NP cells. Eight subpopulations were visualized. **(C)** Distribution of eight NP subpopulations (defined in B) in different groups. **(D)** Heatmap revealing the scaled expression of DEGs for each NP subpopulation. DEGs, differentially expressed genes. **(E)** GO (gene ontology) functions of eight NP subpopulations defined in B. **(F)** Immunofluorescence staining showing the expression of HIF1A, TOMM20, SOD2 in human NP tissues. Scale bar 100 µm. **(G)** Quantification of HIF1A, TOMM20, SOD2 expression.

To further understand the heterogeneity within total NP cells (including NPPC, division-NPPC, and NP cell in Figure 1B), we conducted subpopulation analysis. Using Uniform Manifold Approximation and Projection (UMAP) dimensionality reduction analysis, we divided the total NP cells into 10 clusters (Supplementary Figure S1). GO enrichment analysis suggested significant enrichment of the oxidative phosphorylation pathway in cells from cluster 0 and cluster 5. Since the normal intervertebral disc is avascular tissue, the internal environment is characterized by hypoxia. Studies have demonstrated that HIF1A plays a crucial role in enabling NP cells to adapt to hypoxic conditions and in inhibiting the production of reactive oxygen species (ROS). Conversely, normoxic conditions have been shown to result in a downregulation of *HIF1A* expression in NP cells, which in turn leads to an increase in ROS levels. Furthermore, the reduction of ROS in NP cells under hypoxic conditions is dependent on HIF1A, emphasizing the critical function of HIF1A in the typical survival of NP cells under hypoxic conditions (Silagi et al., 2021; Madhu et al., 2023; Yang et al., 2023; Song et al., 2024). Therefore, combining the expression pattern of HIF1A with the results of GO enrichment analysis, we divided the total NP cells into 8 celltypes, namely, *HIF1A* ^high^_NP1-2, *HIF1A* ^low^_NP1-3, and NPPC1-3 (Figures 2A, B; Supplementary Figure S2). We found that in the NY group, *HIF1A* ^high^_NP2 cells with high expression of *HIF1A* were predominant, which is consistent with previous research indicating that *HIF1A* is highly expressed in NP cells of normal intervertebral discs to adapt to hypoxia. In the NO and MY groups, the main composition consisted of *HIF1A* ^low^_NP1 cells with low expression of *HIF1A*, suggesting that the hypoxic environment within the intervertebral discs may be disrupted in these groups, leading to the suppression of *HIF1A* expression in the NP cells. It is worth mentioning that in the MO group, *HIF1A* ^high^_NP1 accounted for the majority. Although these NP cells had high expression of *HIF1A*, they still exhibited differences from *HIF1A* ^high^_NP2 cells in normal NP on the UMAP, indicating potential differential expression at other molecular levels (Figure 2C).

To further explore the functional differences among subgroups of cells, we calculated the DEGs of each celltype (Figure 2D) and performed GO enrichment analysis. Of note, *HIF1A* ^high^_NP2 showed high expression of *COL2A1* and *NR4A1*. *COL2A1* encodes type II collagen fibers, which increase intervertebral disc stability (Trefilova et al., 2021), while *NR4A1* has been reported to participate in the regulatory process of inhibiting intervertebral disc degeneration (Feng et al., 2017). *HIF1A* ^low^_NP1 cells exhibited high expression of *MMP3* and *SOD2*. MMP3 can degrade the extracellular matrix and is believed to promote intervertebral disc degeneration (Goupille et al., 1998; Roberts et al., 2000; Vo et al., 2013), while SOD2 is a pivotal antioxidant enzyme that eliminates intracellular superoxide and can convert oxygen radicals into hydrogen peroxide, thereby alleviating intracellular oxidative stress (Tamagawa et al., 2024). This suggests the presence of oxidative stress pressure within these cells and their potential contribution to intervertebral disc degeneration. *HIF1A* ^high^_NP1 cells showed high expression of *APOD*, *DNAJB1*, and *HSPB1*. *APOD* encodes apolipoprotein, suggesting a possible alteration in lipid metabolism pathways in these cells, while *DNAJB1* and *HSPB1* are known stress-response characteristic genes. Previous studies have shown that an increase in ROS levels can induce elevated expression levels of these two genes (He et al., 2024). This implies that although *HIF1A* ^high^_NP1 cells have low *HIF1A* expression, there may still be some stress factors present within these cells. GO enrichment analysis (Figure 2E) indicates that *HIF1A* ^high^_NP2 cells are mainly associated with ossification, connective tissue development, and cartilage development. In *HIF1A* ^low^_NP1 cells, pathways related to mitochondrial translation, aerobic respiration, and oxidative phosphorylation were significantly enriched. Previous research has shown that due to the low oxygen environment within the intervertebral disc, normal nucleus pulposus cells primarily rely on glycolysis as their main metabolic mode, and high-intensity oxidative phosphorylation is an abnormal manifestation (Agrawal et al., 2007; Francisco et al., 2023). Considering the low expression level of *HIF1A* in these cells, it suggests the presence of higher oxygen levels in the microenvironment that *HIF1A* ^low^_NP1 cells encounter, enabling them to engage in oxidative phosphorylation. Previous studies have indicated that high-intensity oxidative phosphorylation can increase ROS levels, leading to oxidative stress (Dimozi et al., 2015; Cheng et al., 2022; Excessive reactive oxygen species, 2022). These cells also exhibited high expression of *SOD2*, which may serve as a protective mechanism against high intracellular ROS levels, further reflecting the high ROS levels (Tamagawa et al., 2024).

To further explore the oxidative stress pressure of various subtypes of cells, we used the AUCell to analyze the strength of relevant pathways in different cell populations. The results showed that the hypoxia pathway was weaker in *HIF1A* ^low^_NP1, while the oxidative phosphorylation, ROS, DNA repair, and ferroptosis pathways were stronger. Interestingly, the opposite trend was observed in the *HIF1A* ^low^_NP1 cells for the above-mentioned pathways. It is worth noting that there was no significant difference in the strength of the glycolysis pathway among different cell populations (Supplementary Figure S3A). Since *HIF1A* ^low^_NP1 cells are mainly distributed in MY, with fewer in MO, indicating an upregulation of oxidative stress levels in the NP of young degenerative patients, which is a defining feature of this type of disc degeneration. We performed hematoxylin-eosin staining (H&E stain, Supplementary Figure S3B) and immunofluorescence staining on NP tissue samples from MY and MO patients. The results showed lower HIF1A level and higher TOMM20 level in the MY group, with a patchy distribution suggestive of intense oxidative phosphorylation. SOD2 level increased in the MY group (Figures 2F, G), while COL2A1 level decreased (Supplementary Figures S3C, D). In summary, the above results indicate that NP cells from patients in the NO and MY groups exhibit relatively higher levels of oxidative phosphorylation intensity, resulting in a higher degree of oxidative stress within their cells. Although NP cells from patients in the MO group also express stress response characteristic genes, their pathogenic factors are unrelated to oxidative phosphorylation and instead associated with the unfolded proteins and other stressors, indicating endoplasmic reticulum stress.

### Pseudotime trajectories analysis reveals distinct differentiation trajectories of nucleus pulposus cells

**FIGURE 3:**
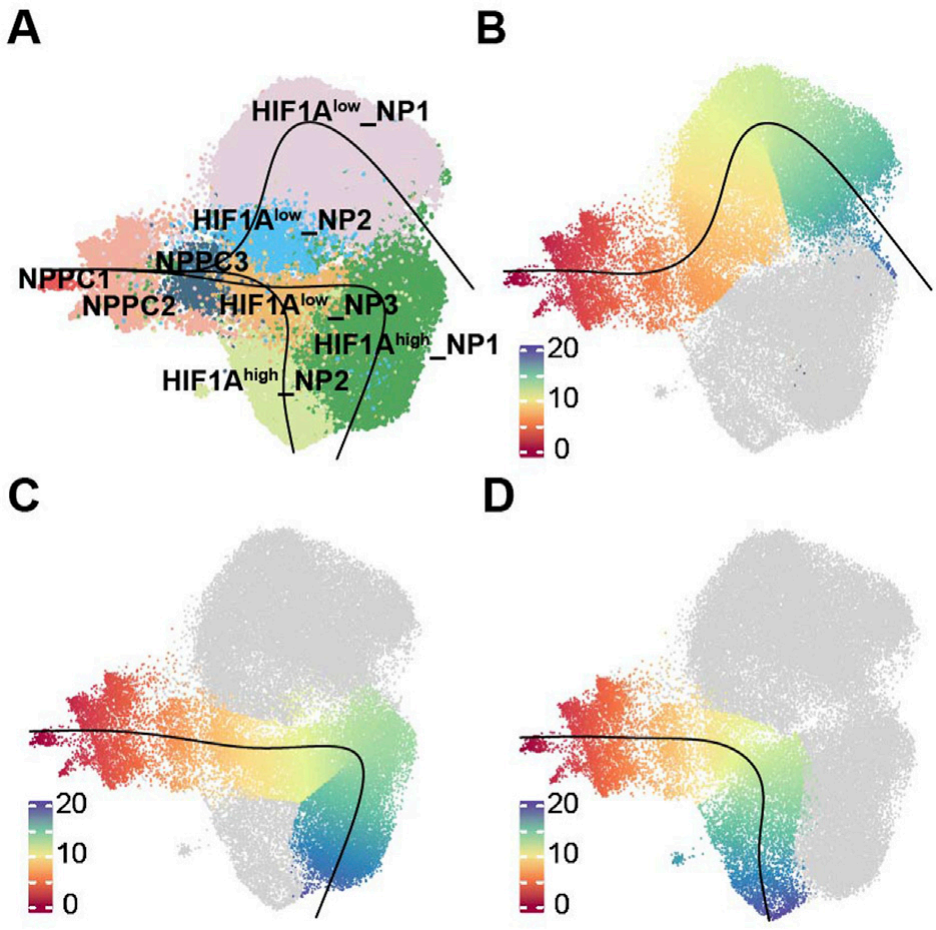
Trajectories of NP subpopulations. **(A)**Slingshot-based pseudotime trajectories inferred from UMAP embedding of NP subpopulations. **(B)** showed the trajectory, NPPC1, NPPC2, NPPC3, HIF1Alow_NP3, HIF1Alow_NP1. **(C)** showed the trajectory, NPPC1, NPPC2, NPPC3, HIF1Alow_NP3, HIF1Ahigh_NP1. **(D)** showed the trajectory, NPPC1, NPPC2, NPPC3, HIF1Alow_NP3, HIF1Ahigh_NP2.

To investigate how different terminally differentiated NP cells differentiate from NPPC, we conducted pseudotime trajectories analysis using Slingshot. Since NPPC1 possesses strong proliferative ability (Figure 2E; Supplementary Figure S4 cell cycle), it is recognized as the most stem-like NP cell and set as the starting point of differentiation. We found three differentiation trajectories in NP cell differentiation (Figures 3A–D), from NPPC1 to NPPC2, further to NPPC3 and *HIF1A* ^low^_NP3, and subsequently branching into three distinct outcomes: *HIF1A* ^low^_NP1, *HIF1A* ^high^_NP1, and *HIF1A* ^high^_NP2. These three types of cells represent the dominant cell components of the NO and MY, MO, and NY groups, respectively. pseudotime trajectories analysis suggests that they also represent terminal differentiation states within their respective groups. Therefore, it can be inferred that NPPC2, NPPC3, and *HIF1A* ^low^_NP3 serve as common transitional states during NPPC1 differentiation into terminally differentiated NP cells. In trajectory 1, *HIF1A* ^low^_NP2 represents a unique transitional state in this fate process. Thus, through pseudotime trajectories analysis, we have identified the differentiation trajectories of representative cells in each group, and their fate determination may depend on the environmental factors encountered by *HIF1A* ^low^_NP3.

### Dendritic cells play an important role in the IVD

**FIGURE 4:**
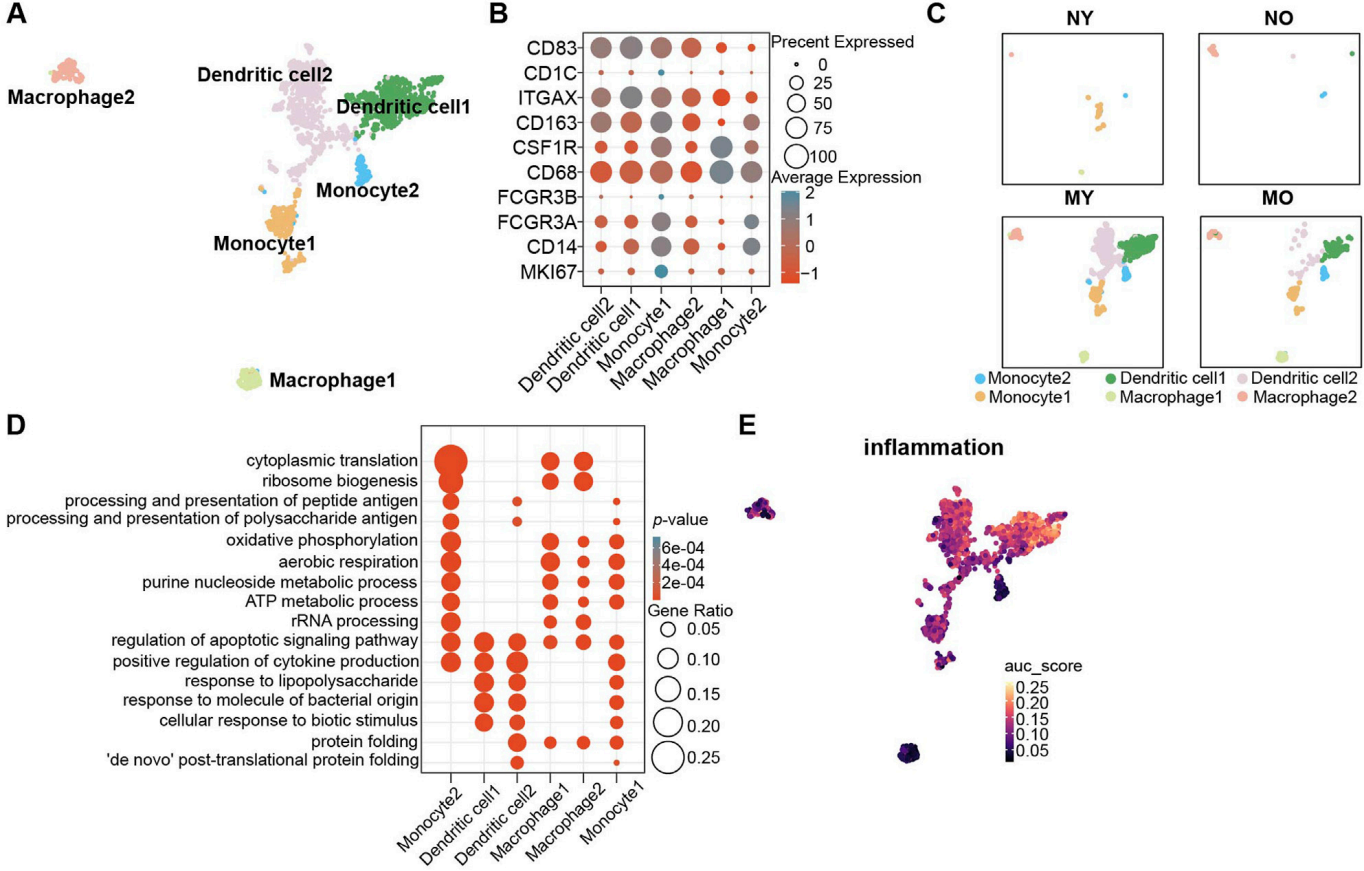
Identification of myeloid cell subpopulations in human IVD and their distribution in different groups. **(A)** UMAP plot showing the single-cell transcriptomic landscape of myeloid cells. Six clusters were visualized. **(B)** Dot plot of marker genes for myeloid cell subpopulations. Color scale indicates the mean of normalized expression of marker genes in each cell type, and dot size is proportional to the percentage of cells within each cell cluster expressing the marker genes. **(C)** Same UMAP visualization as the one in B but split by different groups. **(D)** GO (gene ontology) functions of six cluster defined in **(A)**. **(E)** UMAP visualization AUC scores of the inflammation pathway in myeloid cells.

Due to the avascular nature of intervertebral discs, they are in an immune-privileged state. To explore the pathological and physiological mechanisms of immune cells in different groups, we performed subpopulation analysis of myeloid cells. Based on the classic marker and GO enrichment analysis results, we divided myeloid cells into six celltypes (Figures 4A, B; Supplementary Figure S5), namely, monocytes 1-2 (*CD14*, *FCGR3A*, *FCGR3B*), dendritic cells 1-2 (*ITGAX*, *CD1C*, *CD83*), and macrophages 1-2 (*CD68*, *CSF1R*, *CD163*). We found that myeloid cells were mainly distributed in degenerated groups, and the MY group had more myeloid cells. Among the myeloid cells, dendritic cells were the predominant component (Figure 4C). In monocytes, monocyte 1 exhibited strong proliferative activity (*MKI67*+), while the GO enrichment analysis results for monocyte 2 (Figure 4D) suggested significant enrichment of antigen processing and presentation function, which is an important function of dendritic cells. Although both dendritic cell 1 and 2 showed high expression of classic markers, only dendritic cell 2 had significant enrichment of antigen processing and presentation function, revealing the heterogeneity within dendritic cells. Additionally, we also found significant enrichment of the response to lipopolysaccharide pathway in dendritic cells 1 and 2, suggesting that they exhibit a pro-inflammatory phenotype. The AUCell results indicated a significant increase in the intensity of the inflammation pathway in dendritic cells 1 and 2, while the intensity was relatively low in other myeloid cell populations (Figure 4E).

**FIGURE 5:**
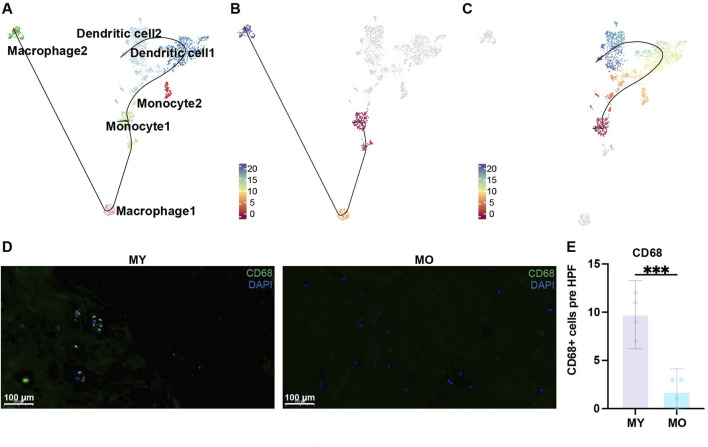
Trajectories of myeloid cell subpopulations. **(A)** Slingshot-based pseudotime trajectories inferred from UMAP embedding of myeloid cell subpopulations. **(B)** showed the trajectory, monocyte1, macrophage1, macrophage2. **(C)** showed the trajectory, monocyte1, dendritic cell1, dendritic cell2. **(D)** Immunofluorescence staining showing the expression of CD68 in human NP tissues. Scale bar 100 µm. **(E)** Quantification of CD68 expression.

Through Slingshot pseudotime analysis, we discovered two differentiation trajectories from monocytes to dendritic cells and monocytes to macrophages (Figure 5A). Monocyte 1 exhibited strong proliferative capacity, so we defined it as the starting point of differentiation. Slingshot pseudotime analysis showed two differentiation trajectories. Monocyte1 differentiated into macrophages 1-2 or monocyte1 differentiated into dendritic cells 1-2 via monocyte 2, which served as a transitional state (Figures 5B, C). This suggests that once monocytes begin to differentiate, their proliferative capacity diminishes, and they start exhibiting immune-related functions, particularly enhanced antigen presentation. This indicates a tendency for monocyte 2 to differentiate into dendritic cells, as supported by the higher proportion of dendritic cells in this group, confirming the accuracy of this differentiation trajectory. Dendritic cell 1 represents the transitional state from monocyte 2 to dendritic cell 2. Interestingly, during this transitional state, antigen presentation functionality decreases but reappears in the terminally differentiated dendritic cell 2 (Figure 4D). The role of dendritic cells is to present antigens through MHC-II molecules to facilitate antigen recognition by CD4^+^ T cells and initiate downstream immune responses. In the immune-privileged state of the intervertebral disc, immune cell infiltration may lead to the recognition of self-antigens present in the NP as foreign substances. Consequently, there is a tendency for a significant differentiation into dendritic cells to meet the demand for antigen presentation. We found more CD68 positive cell in MY group with immunofluorescence staining, which is consist with our single-cell sequencing analysis results (Figures 5D, E). Therefore, in the NP of MY group, dendritic cells are involved in antigen presentation of internal antigens and exhibit pro-inflammatory responses.

### Cellular communication analysis reveals the interactions between various cell types in different groups

**FIGURE 6:**
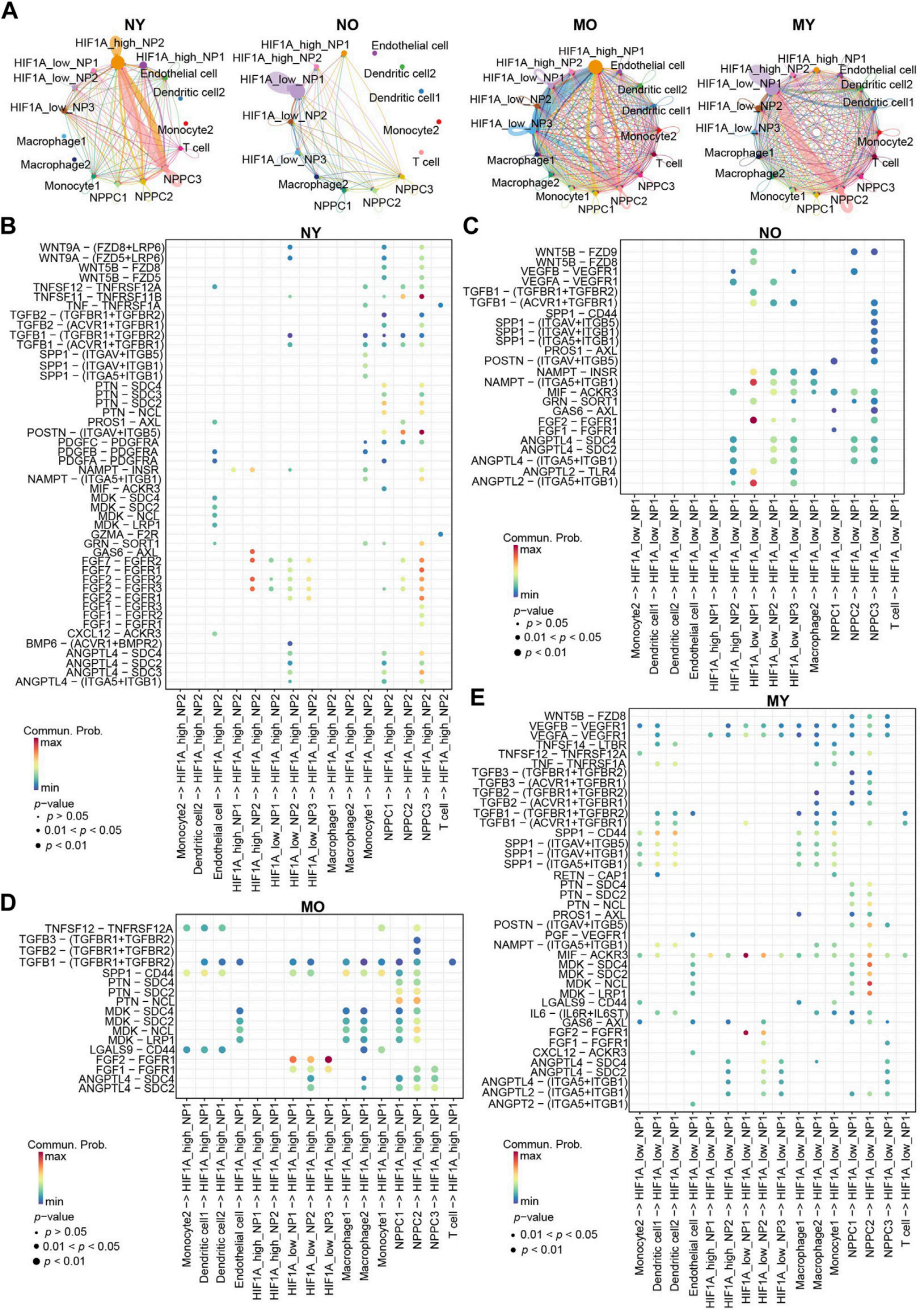
Overview of the crosstalk networks among the clusters of human IVD cells. **(A)** The intercellular crosstalk networks among NP cells and myeloid cells in different groups. **(B–E)** Dot plot showing the communication probability of the indicated ligand-receptor pairs between NP cell subpopulations and myeloid cells subpopulations in different groups.

To further understand the differences in cellular communication between the groups, we used CellChat to analyze cellular communication among all cell types in the four groups (Figure 6A). The results showed that within the *HIF1A* ^high^_NP2 subgroup of the NY group, there is a strong mutual communication between NPPC3 and *HIF1A* ^high^_NP2. In the NO group, self-communication exists only within the *HIF1A* ^low^_NP1 subgroup. In the MO group, communication mainly occurs between *HIF1A* ^high^_NP1, *HIF1A* ^low^_NP3, and NPPC2. In the MY group, there is communication of varying intensities between *HIF1A* ^low^_NP1, NPPC2, and dendritic cells 1-2.

Bubble plots display communication within the NY group (Figure 6B). *HIF1A* ^high^_NP2 interacts with NPPC3 through FGF2/FGFR2. FGF2/FGFR2 plays a crucial role in various important biological processes such as cell survival, proliferation, differentiation, and migration. We speculate that this may be a mechanism maintaining normal homeostasis in the NY group. Additionally, GAS6/AXL communication also exists within *HIF1A* ^high^_NP2, which is a classic efferocytosis mechanism widely found in the process of macrophage engulfing apoptotic cells (Bellan et al., 2019; Vago et al., 2021). Previous studies have indicated that NP cells possess certain phagocytic ability (Nerlich et al., 2002; Jones et al., 2008; Lin et al., 2019). Hence, we hypothesize that the senescent NP cells within the NY group, lacking immune cells, are likely engulfed by the healthy NP cells through the process of phagocytosis, thereby maintaining internal homeostasis. It is noteworthy that POSTN/(ITGAV + ITGB5) and TNFSF11/TNFRSF11B represent the most significant modes of communication in the NY group, primarily occurring between NPPC3 and *HIF1A* ^high^_NP2 cells. In the NO group (Figure 6C), ANGPTL2/(ITGA5 + ITGB1), FGF2/FGFR1, and NAMPT/(ITGA5 + ITGB1) communication is predominantly observed within *HIF1A* ^low^_NP1 cells. In the MO group (Figure 6D), the main mode of communication for *HIF1A* ^low^_NP1-3 is through FGF2/FGFR1 with HIF1Ahigh_NP1 cells. In the MY group (Figure 6E), the most significant modes of communication are FGF2/FGFR1 and MIF/ACKR3 within *HIF1A* ^low^_NP1. Ligand MDK and receptors LRP1, NCL, SDC2, and SDC4 mediate communication between NPPC2 and *HIF1A* ^low^_NP1. Furthermore, dendritic cells 1-2 secrete SPP1 protein that acts on multiple receptors of *HIF1A* ^low^_NP1 cells. *HIF1A* ^low^_NP1 cells can also affect dendritic cells 1-2 through MIF acting on CD74, CXCR4, and CD44 receptors (Figure 3). This suggests a strong immunomodulatory effect between dendritic cells and *HIF1A* ^low^_NP1 within the MY group. Additionally, *HIF1A* ^low^_NP1 can act on endothelial cell VEGFR through VEGFA, potentially leading to vascular formation within the intervertebral disc and disrupting its hypoxic environment. In summary, we have discovered distinct characteristics of cellular communication among different groups. These communication modes suggest potential molecular mechanisms for maintaining homeostasis in NP cells and the changes that occur in communication between cell types when homeostasis is disrupted under different circumstances.

## Discussion

Intervertebral disc degeneration is often thought to occur mainly in the elderly population. However, there is a gradual increase in the proportion of young patients (Liu et al., 2023). In recent years, single-cell sequencing has been increasingly used in the field of intervertebral disc degeneration (Wang et al., 2023; Jiang et al., 2022; Guo et al., 2023b; Gao et al., 2022; Gan et al., 2021). However, most studies have focused on exploring the severity of degeneration or disc development, without investigating the differences between intervertebral disc degeneration in different age groups. In order to determine whether there are differences in degenerative mechanisms between young and elderly patients, we collected single-cell sequencing data from intervertebral discs of 10 cases, including Normal Young (NY), Normal Old (NO), Mild Young (MY), and Mild Old (MO) types based on the Pfirrmann classification. We conducted a single-cell resolution analysis to elucidate the differences between young and elderly degenerative patients.

During the aging process, the proportion of nucleus pulposus progenitor cells (NPPCs) decreases, indicating a depletion of stem cells. We subdivided NP cells based on the expression levels of hypoxia-inducible factor 1 alpha (*HIF1A*) and identified a subset in young patients with low *HIF1A* expression and aerobic respiration, which we termed *HIF1A* ^low^_NP1. It is worth noting that this cell type is predominant in the intervertebral discs of normal elderly individuals. Previous studies have shown that NP cells primarily metabolize through glycolysis to adapt to the hypoxic environment in the disc center. It has been indicated that under hypoxic conditions, *HIF1A* expression in NP cells increases, leading to the inhibition of reactive oxygen species (ROS) generation. Conversely, normoxic conditions induce downregulation of *HIF1A* expression in NP cells, resulting in increased ROS levels. Furthermore, under hypoxic conditions, the decrease in ROS in NP cells is reliant on *HIF1A*, emphasising the crucial function of *HIF1A* in the typical survival of NP cells under hypoxic conditions (Peng et al., 2024).

*HIF1A* ^low^_NP1 cells are the primary component in both young degenerative patients and normal elderly disc nucleus pulposus cells. This suggests that the anaerobic environment within their intervertebral discs may be disrupted. Previous studies have shown that oxidative phosphorylation can induce ROS generation (Zhang et al., 2022). Elevated levels of oxidative phosphorylation within *HIF1A* ^low^_NP1 cells may lead to increased intracellular ROS generation, inducing oxidative stress damage. High expression of SOD2 and MMP3 was observed in this cell type. SOD2 is an important antioxidant enzyme that clears intracellular superoxide (Bolduc et al., 2019), and its upregulation indirectly indicates elevated intracellular oxidative stress levels. MMP3 can degrade extracellular matrix components, including various types of collagen, and is believed to promote disc degeneration (Lerner et al., 2018). Immunofluorescence staining confirmed a decrease in type II collagen in young patients, which may be associated with the high expression of MMP3 in *HIF1A* ^low^_NP1 cells. This reduction in extracellular matrix weakens the disc’s resistance to mechanical pressure, exacerbating cell death within the disc and promoting degeneration (Hu et al., 2020).

Intercellular communication indicates that *HIF1A* ^low^_NP1 cells can influence endothelial cells through *VEGFA*, promoting angiogenesis within the disc and disrupting the hypoxic environment. Therefore, the *HIF1A* ^low^_NP1 cell population is a significant characteristic of intervertebral disc degeneration in young patients, and these cells may contribute to disc degeneration. In contrast, elderly patients exhibit a predominance of *HIF1A* ^high^_NP1 cells within the intervertebral disc. Although these cells share high *HIF1A* expression with normal NP cells, functional enrichment analysis suggests that they respond to unfolded proteins and other stressors, indicating endoplasmic reticulum stress (Di Conza et al., 2023). Previous studies have also shown that endoplasmic reticulum stress is a significant pathological feature of disc degeneration, leading to apoptosis of NP cells (Liao et al., 2019).

To summarise, intervertebral disc degeneration in young patients is characterised by oxidative stress, whereas in elderly patients, endoplasmic reticulum stress is a significant feature.

The study found that immune cells were only present in degenerative samples, while they were absent in the intervertebral discs of normal and elderly populations. This is consistent with previous reports of the disc being an immune-privileged organ (Sun et al., 2020; Ye et al., 2022). The sequencing data and immunofluorescence staining results indicated a higher proportion of immune cells in young patients compared to elderly patients, which suggests a potential role for immune cells in young patients. Previous studies have indicated that autoimmunity may play an important role in IVDD. In these circumstances, the autoimmune disorder commences following the exposure of antigenic disc components to the circulating blood, resulting in abnormalities in cell-mediated and humoral autoimmune responses and the production of chronic inflammation with low back pain and sciatica (Di Martino et al., 2013). Monocytes within the myeloid cell subset are more likely to differentiate into dendritic cells than macrophages, although both can represent antigen-presenting cells in the process of autoimmune response. Dendritic cells present antigen to naïve CD4^+^ T cells during the initiation of an immune response (Geiss et al., 2016), whereas macrophages display antigen to previously activated CD4^+^ T cells at a later stage of the manifested immune response. Additionally, dendritic cells display a stronger pro-inflammatory phenotype (Lan et al., 2022). Studies have shown that a culture medium that favours M1 macrophages can promote intervertebral disc degeneration in rats. This can induce NP cells to express MMP13 and inflammatory factors, suggesting that pro-inflammatory immune cells may have adverse effects on NP cells (Li et al., 2022). Therefore, the pro-inflammatory dendritic cells in this study may further promote disc degeneration. As previously stated, the infiltration of immune cells in the NP area is closely related to the formation of nerves and blood vessels, which may result in an interaction between the NP and the blood circulation. Thus, a recent study has demonstrated that the circulating levels of cartilage oligomeric matrix protein (COMP) and a disintegrin and metalloproteinase with thrombospondin motifs 7 (ADAMTS7) can be examined as promising indicators to detect IVDD (Ding et al., 2024). These biomarkers may help us better dignose IVDD at an early stage.

Future research should aim to elucidate the following points. Why do young and elderly patients present different pathological characteristics in intervertebral disc degeneration? The pathogenic mechanisms in young patients are unclear. Can inhibiting oxidative stress or endoplasmic reticulum stress improve disc degeneration, and what are the key mechanisms and targets? Finally, this project leaves unanswered questions about why monocytes differentiate into dendritic cells and the specific roles that dendritic cells play in intervertebral disc degeneration. These questions require further exploration in future studies.

## Data Availability

The data presented in the study are deposited in the GEO database, accession number (GSE233666, GSE205535, GSE189916), and in the CNGBdb database accession number CNP0002664 available via the following link: https://db.cngb.org/search/project/CNP0002664/.

## Supplementary material

SUPPLEMENTARY FIGURE S1 UMAP visualization showing the human IVD cells were divided into 10 clusters.

SUPPLEMENTARY FIGURE S2 Violin polt showing HIF1A expression in different NP cell subpopulations. Using wilcoxon test, *p* < 0.0001 (****).

SUPPLEMENTARY FIGURE S3
**(A)** Strength of the glycolysis pathway among different cell populations. **(B)** H&E staining on NP tissues. **(C)** Immunofluorescence staining showing the expression of COL2A1 in human NP tissues. Scale bar 100 µm. **(D)** Quantification of COL2A1 expression.

SUPPLEMENTARY FIGURE S4
**(A)** Bar graph representing the proportion of each cell cycle stage in 8 NP cell subpopulations. **(B)** Bar graph representing the amount of cells in 8 NP cell subpopulations.

SUPPLEMENTARY FIGURE S5
**(A)** UMAP visualization showing the immune cells were divided into 6 clusters.

SUPPLEMENTARY FIGURE S6
**(A)** The sagittal and horizontal views of the computed tomography image serve to illustrate the area that was chosen for the immunofluorescence procedure. **(B)** A schematic diagram of the disc represent the area that was chosen for the immunofluorescence.
