# Supplementary figures and images for: Investigating the characteristics of mild intervertebral disc degeneration at various age stages using single-cell genomics
Source: Front Cell Dev Biol. 2024 Jul 2;12:1409287. doi: 10.3389/fcell.2024.1409287 (PMC11250600; doi:10.3389/fcell.2024.1409287)

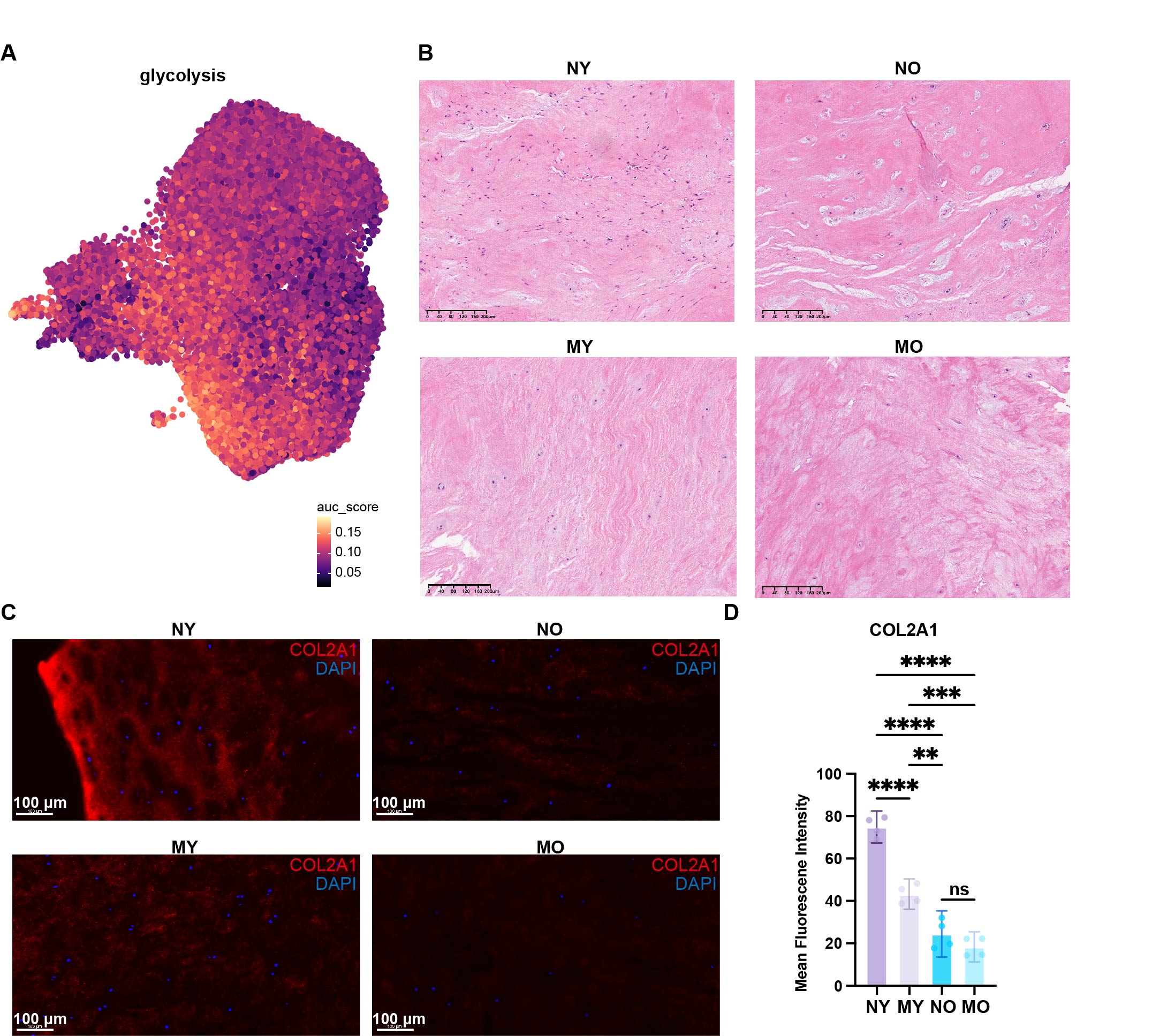

Supplement: Supplementary file 1 [file Image3.JPEG]

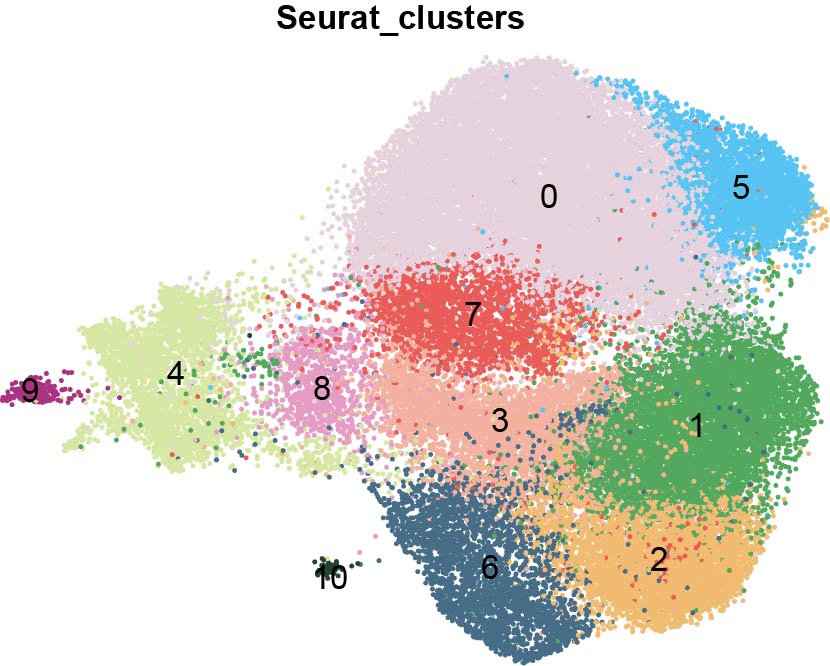

Supplement: Supplementary file 3 [file Image1.JPEG]

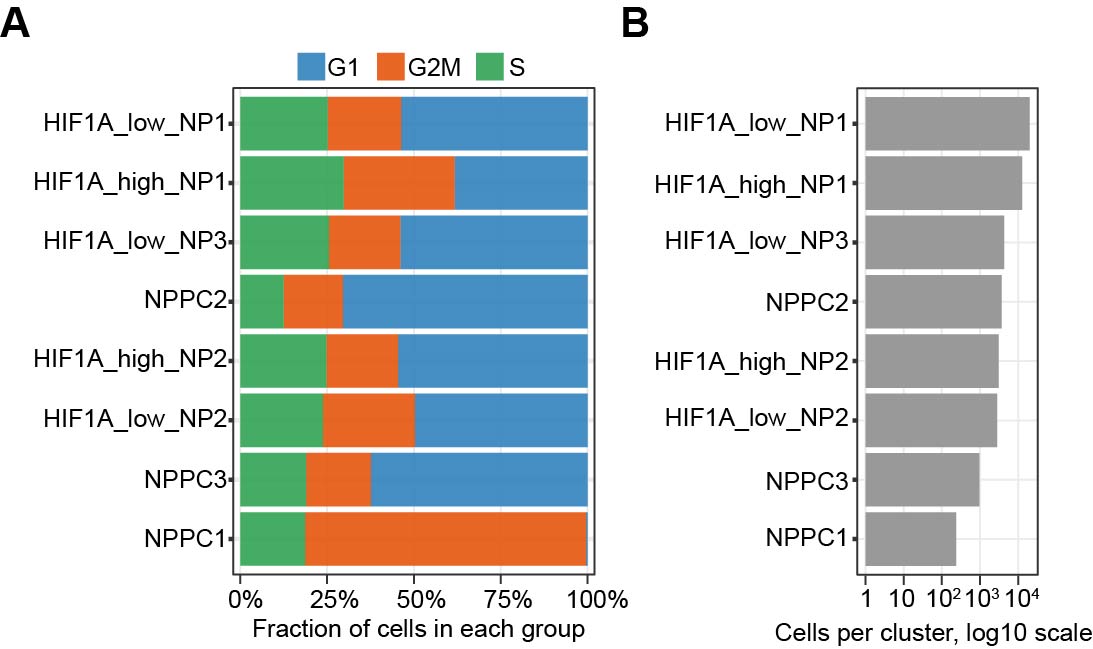

Supplement: Supplementary file 4 [file Image4.JPEG]

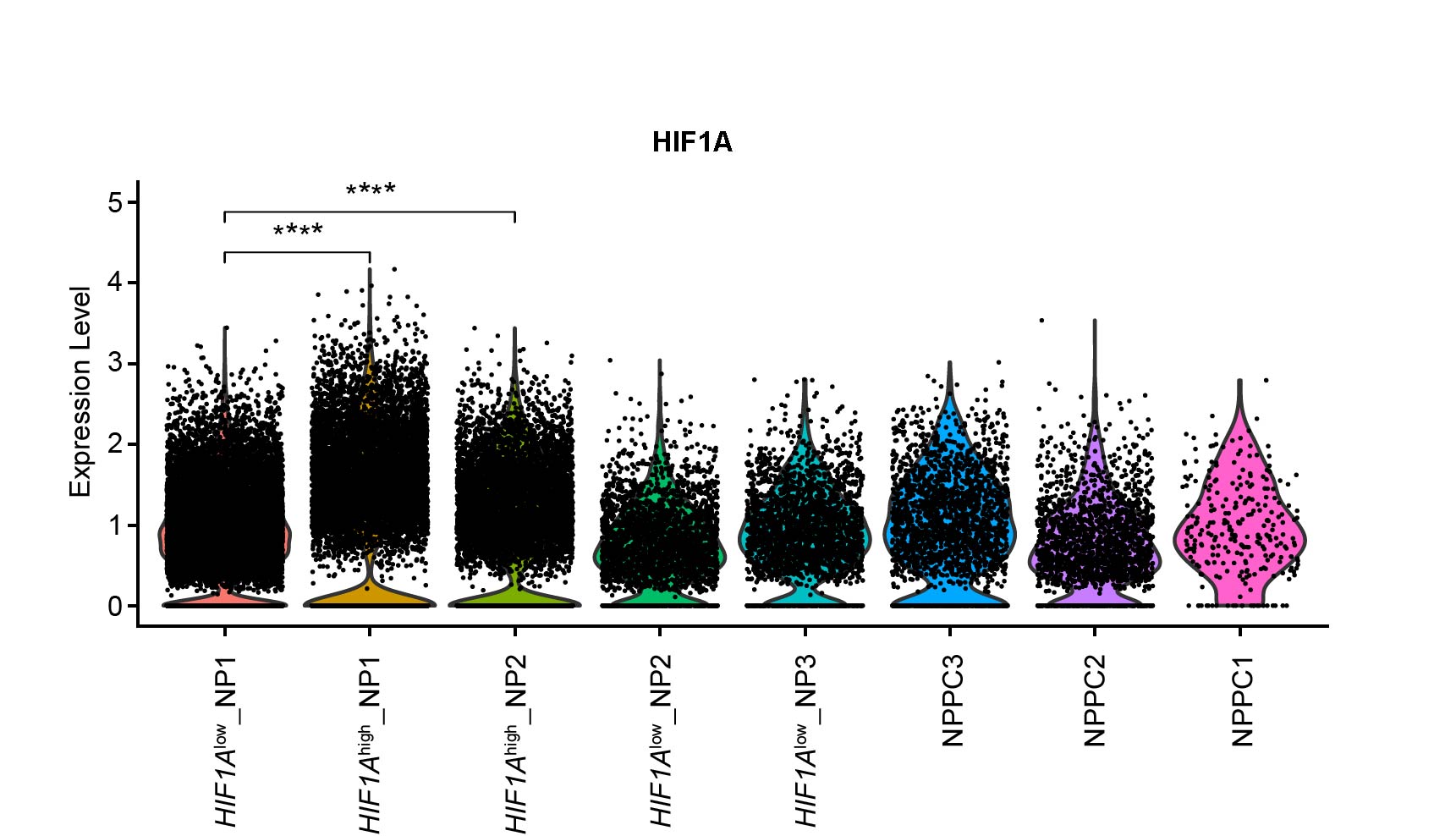

Supplement: Supplementary file 5 [file Image2.JPEG]

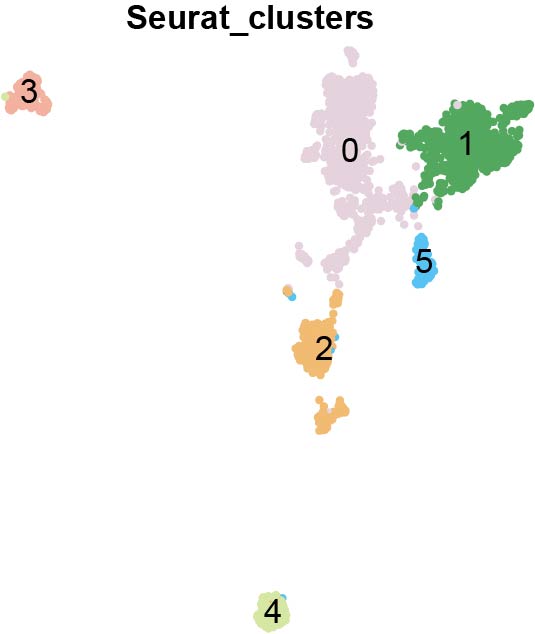

Supplement: Supplementary file 6 [file Image5.JPEG]

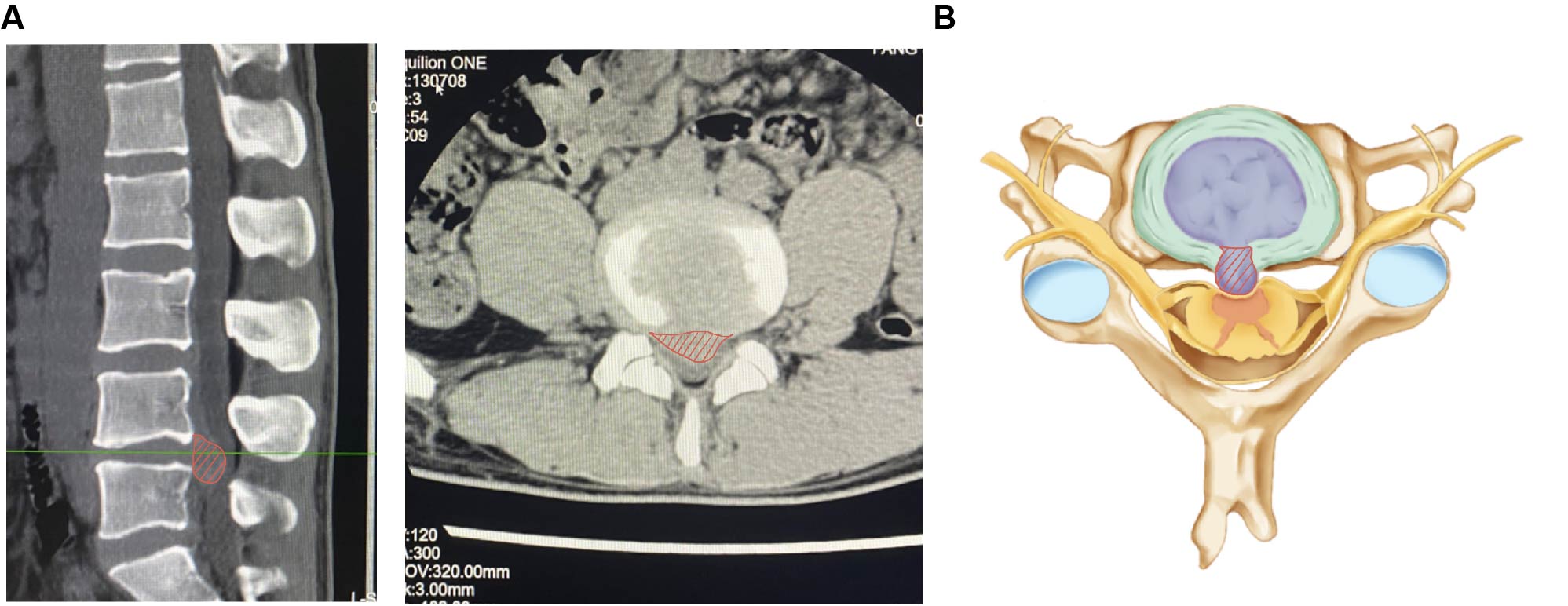

Supplement: Supplementary file 8 [file Image6.JPEG]
