# Supplementary material for: Investigating the characteristics of mild intervertebral disc degeneration at various age stages using single-cell genomics
Source: Front Cell Dev Biol. 2024 Jul 2;12:1409287. doi: 10.3389/fcell.2024.1409287 (PMC11250600; doi:10.3389/fcell.2024.1409287)
Supplement: Supplementary file 2 [file Table1.DOCX]

Table S1 Characteristics of the samples collected from surgery

| Group | Age | Gender | Location | Pfirrmann Grade | Complication | |
| --- | --- | --- | --- | --- | --- | --- |
| NY | 21 | Male | L4/L5 | 1 | | No |
| NY | 28 | Male | L3/L4 | 1 | | No |
| NY | 27 | Female | L4/L5 | 1 | | No |
| NY | 22 | Male | L4/L5 | 1 | | No |
| MY | 27 | Female | L4/L5 | 3 | | No |
| MY | 20 | Male | L4/L5 | 2 | | No |
| MY | 23 | Male | L4/L5 | 3 | | No |
| MY | 21 | Female | L5/S1 | 3 | | No |
| NO | 62 | Male | L3/L4 | 1 | | No |
| NO | 73 | Male | L3/L4 | 1 | | No |
| NO | 68 | Female | L4/L5 | 1 | | No |
| NO | 70 | Female | L4/L5 | 1 | | No |
| MO | 72 | Female | L4/L5 | 3 | | No |
| MO | 68 | Male | L5/S1 | 3 | | No |
| MO | 65 | Female | L4/L5 | 3 | | No |
| MO | 66 | Male | L4/L5 | 3 | | No |
