# Supplementary material for: Investigating the characteristics of mild intervertebral disc degeneration at various age stages using single-cell genomics
Source: Front Cell Dev Biol. 2024 Jul 2;12:1409287. doi: 10.3389/fcell.2024.1409287 (PMC11250600; doi:10.3389/fcell.2024.1409287)
Supplement: Supplementary file 7 [file Table2.DOCX]

Table S2 Characteristics of the samples retrieved from the GEO database

| **Sample** | **Group** | **Degeneration Grade** | **Age** | **Gender** | **Database ID** |
| --- | --- | --- | --- | --- | --- |
| MY1 | mild-young | II | 20 | Female | GSE233666 |
| MY2 | mild-young | II | 28 | Female | GSE233666 |
| MY3 | mild-young | III | 23 | Male | GSE233666 |
| MO1 | mild-old | III | 59 | Male | CNP0002664 |
| MO2 | mild-old | III | 56 | Male | CNP0002664 |
| MO3 | mild-old | III | 69 | Female | GSE233666 |
| NY1 | normal-young | I | 11 | Unknown | GSE205535 |
| NO1 | normal-old | I | 65 | Unknown | GSE189916 |
| NO2 | normal-old | I | 65 | Unknown | GSE189916 |
| NO3 | normal-old | I | 65 | Unknown | GSE189916 |
